# Publication rate of abstracts presented at the Canadian society of otolaryngology- head and neck surgery annual meetings: a five year study 2006–2010

**DOI:** 10.1186/s40463-014-0051-5

**Published:** 2014-12-17

**Authors:** Lauren N Ogilvie, Julie Pauwels, Neil K Chadha, Frederick K Kozak

**Affiliations:** Division of Pediatric Otolaryngology, BC Children’s Hospital, 4480 Oak Street, Vancouver, BC V6H 3V4 Canada

**Keywords:** Publication, Otolaryngology, Scientific Meeting

## Abstract

**Background:**

To determine the rate of publication in a peer-reviewed journal for all oral presentations made at the Canadian Society for Otolaryngology- Head and Neck Surgery’s Annual Meetings from 2006–2010.

**Methods:**

All abstracts were searched by keywords and authors’ names in Medline via PubMed and Google Scholar. Authors of presented abstracts not found to be published were contacted directly for further information.

**Results:**

50.5% of presented abstracts (n = 198) were subsequently published with an average time to publication of 21 months. For those abstracts found not to be published 74.6% (n = 167) of authors responded with further information about their research, 66% (n = 89) of abstracts with author response that were not published were never submitted for publication. Authors’ main reasons for not publishing were that the research was still in process (34%, n = 21) or that a resident or fellow working on the project “had moved on” (26%, n = 16).

**Conclusion:**

The publication rate for the Canadian Society for Otolaryngology- Head and Neck Surgery’s Annual Meetings from 2006–2010 is within the range reported by other conferences and specifically other Canadian conferences in different specialties; however, roughly half of presentations went on to be published. The main barrier to publication was bringing projects to the submission stage and not rejection by journals. Resources such as more time for research or personnel to coordinate projects may result in a greater rate of project completion.

## Background

The presentation of research findings at academic meetings is an important form of dissemination. Academic meetings and particularly those that cater to the medical profession must be held to a high standard as research presented may affect the future practice of clinicians in attendance. One of the accepted measures of quality is the ability of presenters to publish their research in peer-reviewed journals; therefore, previous work has been performed to determine publication rates from various academic meetings. Research from around the world and across medical disciplines has reported a range of publication rates from 27-81%, influenced by whether the presentation was a poster or from the podium, the specialty of the research and the study design [1-5].

The mean time from presentation to publication has been reported to range from 15.8-25.3 months; however, a study on the Canadian Association of Pediatric Surgeons and the American Pediatric Surgery Association found that 93% of abstracts were published within 1-year of presentation [3,6-13].

Previously described justification for not publishing include “not seeing publication as a priority,” “not having enough time” and rejection [2,14]. Canadian evaluations of presentation publication in pediatric surgery, plastic surgery and anesthesia have reported publication rates from 45%-65% [7,13,15]. Otolaryngology publication rates have varied with the American Academy of Otolaryngology reporting 32% and later 50% while the UK Otorhinolaryngological Research Society (ORS) reports 69% [5,11,16].

The publication rate of presented abstracts at the largest meeting of Otolaryngologists in Canada, the Annual Meeting of the Canadian Society of Otolaryngology- Head Neck Surgery (CSO-HNS), has not previously been reviewed. Quality assurance of this nature is essential to ensuring the value of presentations at an academic meeting. This review will determine the rate of publication for all abstracts presented in oral form at the CSO-HNS Annual Meetings from 2006–2010.

## Methods

All abstracts published in the CSO-HNS scientific programs for the Annual Meetings from 2006–2010 under the heading “Paper Session” were retrospectively reviewed for publication. We did not include abstracts from the Poliquin Resident’s competition as full manuscripts are submitted in advance for each competitor’s presentation. The initial review was accomplished by searching key words and all authors’ names from each abstract in PubMed and then in Google Scholar, using the full title from each abstract. A presentation abstract was considered published if there was an article identified in a peer-reviewed journal that matched the subject matter, methods, and at least two of the authors listed in the scientific program. This allowed for the possibility of modifications to the study from the time of presentation to the time of publication. Information about each abstract’s sub-specialty and city of origin was obtained from the corresponding meeting’s scientific program.

For abstracts where no publications were identified, the lead authors were contacted to provide the status of their research. If the presented research was confirmed as never published, authors were asked to provide reasons for not publishing. If the contacted author was able to produce evidence of publication, the presented abstract was considered published for our analysis.

## Results

**Figure 1:**
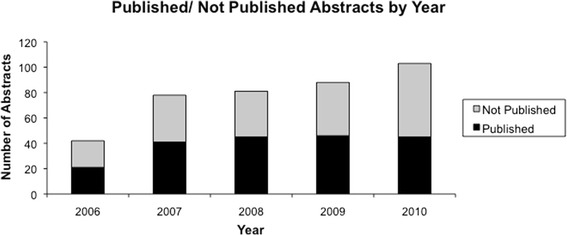
**Number of abstracts that were published/not published for each conference year.**

During the 2006–2010 Annual Meetings for the CSO-HNS 392 abstracts were presented as a part of “Paper Sessions” with an increase in number of presentations by year (see Figure 1). Of these abstracts, 198 (50.5%) were subsequently published in peer-reviewed journals. Average time to publication from presentation was 21 months, with a range of 61 months prior to presentation to 76 months after presentation. In 13 cases (6.6%) the research was published before the conference presentation. Exclusion of these 13 abstracts from analysis resulted in an average time to publication of 24 months.

Canadian researchers submitted the majority of abstracts with each meeting including 2 to 11 submissions from international authors. The most contributions from all five conferences were made by researchers from Montreal, QC (68), Toronto, ON (63) and London, ON (42). The best publication rate of the top seven contributing cities was from London, Ontario (73.8%) and the lowest from Ottawa, Ontario (32%).

**Figure 2:**
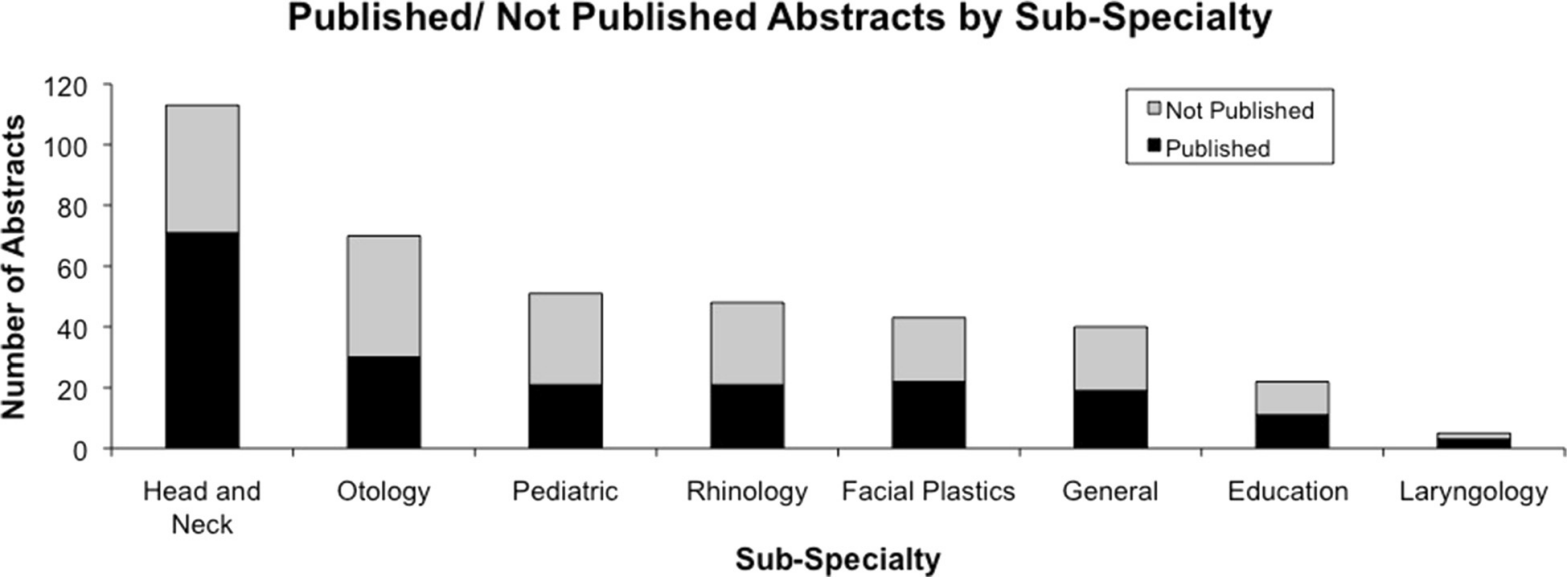
**Number of abstracts that were published/not published for each sub-specialty.**

When compiling the data from all five years, presented abstracts in the sub-specialty “Head and Neck” were the most prevalent (111) and had the highest publication rate (62.8%). “Facial Plastic Surgery” was the next most presented sub-specialty and had a publication rate (51.2%), this was not found to be significantly different from Head and Neck (p = 0.62; X^2^ test). Pediatric presentations had the lowest publication rate at 41.2% (p < 0.05; X^2^ test). The fewest abstracts were submitted under the sub-specialty “Laryngology” as it was not a listed sub-specialty until the 2010 meeting and therefore not included in the determination of the highest publication rate (see Figure 2).

**Figure 3:**
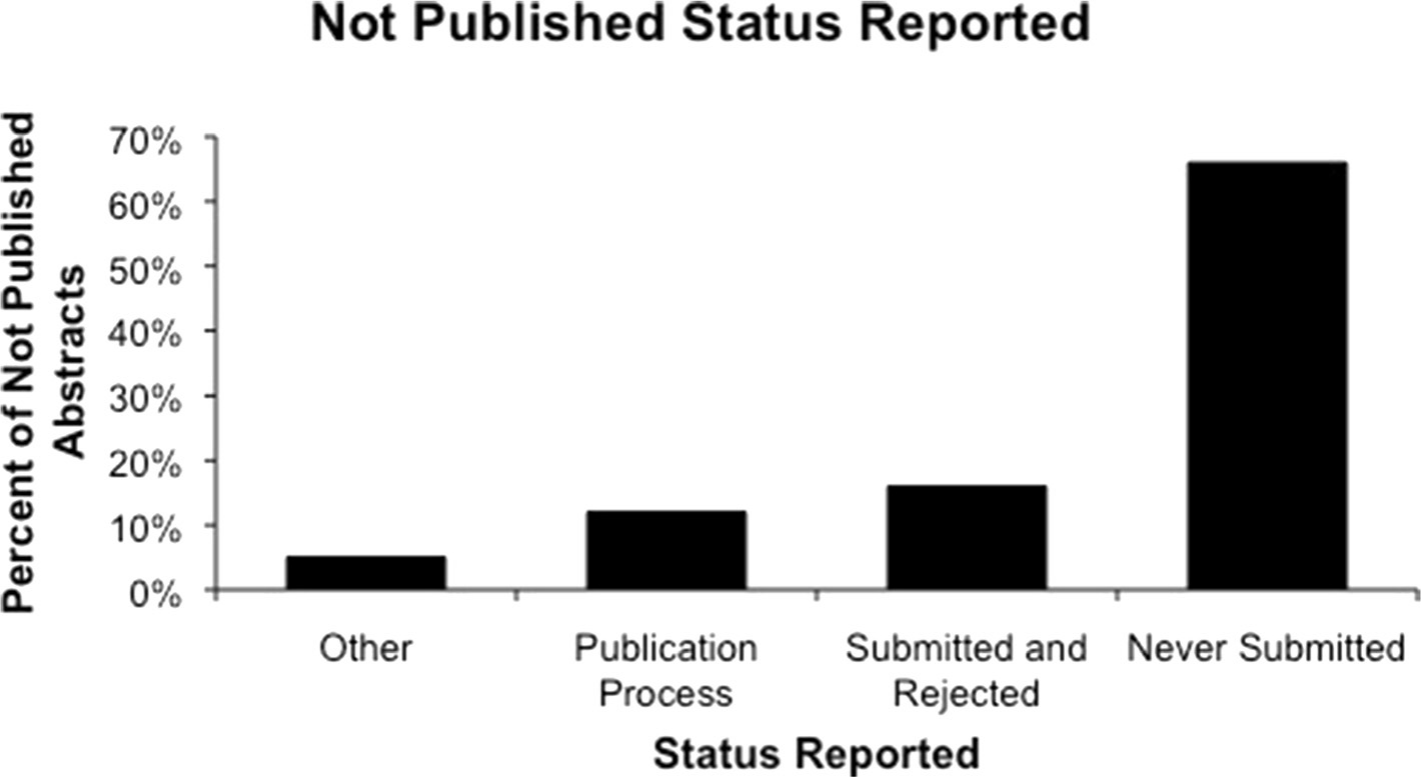
**Reported status of abstracts not published.**

Authors of abstracts determined to be unpublished were asked about the status of their research, and responses were obtained for 167 (74.6%) of these abstracts (see Figure 3). The most common reason for unpublished abstracts was never submitting a manuscript for publication (n = 89, 66.4%), For those who provided further details of reasons for never being submitted (n = 62), in 21 (34%) instances the research was still in progress and 16 (26%) abstracts were not submitted because residents or fellows had moved on. Other reasons given were that similar research was already published, that the research was not substantial enough to be published as a full manuscript, funding to complete the project was lost or that the researchers did not have enough time.

Abstracts that did go on to be published were most often found in the Journal of Otolaryngology-Head and Neck Surgery (n = 87, 46%) and the Laryngoscope (n = 21, 11%). Other journals included JAMA Otolaryngology Head and Neck Surgery (formerly Archives in Otolaryngology-Head and Neck Surgery) (n = 9, 5%), Head and Neck (n = 8, 4%) and the American Journal of Rhinology and Allergy (n = 4, 2%).

## Discussion

This study determined the publication rate of abstracts presented at the Annual Meetings of the CSO-HNS from 2006–2010 to be 50.5%. This rate is within the range previously reported by other medical disciplines and it is within the range (45%-65%) of other Canadian health-related meetings [7,13,15]. This is still considered to be a lower rate as roughly half of abstracts presented went on to publication [17]. The lowest rate of publication was observed in 2010, which may be because it is the most recent conference, however, time to publication is rarely expected to be longer than 4 years after presentation [18].

The sub-specialty of “Head and Neck” had the greatest number of submissions and one of the highest publication rates. Pediatric presentations had the lowest rate of publication, however, this category did include the third lowest number of presentations over these five years. The low publication rate may be due to a lack of time and personnel working in this area. Interestingly, a similar study (American Academy of Otolaryngology Head and Neck Surgery) found that pediatric otolaryngology had the highest rate of publication for their meeting [5].

Previous publication reviews found that common barriers to publishing were “not enough time for research” or “research is not a high priority” [19,20]. This was rarely found in our results and may reflect an academic culture that puts high importance on research. The most common reason for not submitting a manuscript in our study was that the research was still in progress (34%) which may be taken as an indication that there is limited time for research. This result may also indicate that unfinished work was being submitted and presented at the national meeting.

The second most common reason for un-submitted work (26%) was attributed to a resident or fellow moving on from the project. This is possibly due to the transitional nature of these positions; research is often started and not completed to publication. Many residency programs require that residents present research at a conference or research day, but may not emphasize publication. This difficulty has been recognized in Canadian pharmacy residents, where the rate of manuscript publication for projects started in this program was 21% [21]. Encouraging residents to complete studies to publication may be an area in which residency programs and researchers need to pay more attention. Tools such as research timelines and coordinating support have been shown to increase the chance of project completion and subsequent publication in a residency program [22].

The present study was limited in its confirmation of publication as not all journals are included in the Medline database and on Google Scholar. Contacting the presenters of research believed to have not been published added accuracy to our results as authors could provide a citation if their research had in fact been published. Once evidence of publication was given the article was assumed to contain similar research data to what was originally presented. A greater number of responses from researchers about why their work was not submitted for publication would have added to the validity of our survey results.

## Conclusion

Studies of this nature often highlight the importance of ensuring quality in academic meetings. By looking further into the reasons that authors did not publish it is evident that a lack of time or a resident or fellow’s participation in research may result in projects not being completed to publication; however, it can be asserted that certain projects would not be started if it were not for the resident or fellow’s role. It is important that attendees of the CSO-HNS Annual Meetings understand that approximately one half of the presented abstracts do not regularly result in publication. This awareness alone may help researchers better plan for research projects and place a higher emphasis on encouraging residents or fellows to complete research projects to publication.
